# The influence of abrupt increases in seawater *p*CO_2_ on plankton productivity in the subtropical North Pacific Ocean

**DOI:** 10.1371/journal.pone.0193405

**Published:** 2018-04-25

**Authors:** Donn A. Viviani, Daniela Böttjer, Ricardo M. Letelier, Matthew J. Church

**Affiliations:** 1 Department of Oceanography, School of Ocean and Earth Science and Technology, University of Hawaiʻi at Mānoa, Honolulu, HI, United States of America; 2 Daniel K. Inouye Center for Microbial Oceanography: Research and Education, University of Hawaiʻi at Mānoa, Honolulu, HI, United States of America; 3 College of Earth, Ocean, and Atmospheric Sciences, Oregon State University, Corvallis, OR, United States of America; Universidade de Aveiro, PORTUGAL

## Abstract

We conducted a series of experiments to examine short-term (2–5 days) effects of abrupt increases in the partial pressure of carbon dioxide (*p*CO_2_) in seawater on rates of primary and bacterial production at Station ALOHA (22°45’ N, 158° W) in the North Pacific Subtropical Gyre (NPSG). The majority of experiments (8 of 10 total) displayed no response in rates of primary production (measured by ^14^C-bicarbonate assimilation; ^14^C-PP) under elevated *p*CO_2_ (~1100 μatm) compared to ambient *p*CO_2_ (~387 μatm). In 2 of 10 experiments, rates of ^14^C-PP decreased significantly (~43%) under elevated *p*CO_2_ treatments relative to controls. Similarly, no significant differences between treatments were observed in 6 of 7 experiments where bacterial production was measured via incorporation of ^3^H-leucine (^3^H-Leu), while in 1 experiment, rates of ^3^H-Leu incorporation measured in the dark (^3^H-Leu_Dark_) increased more than 2-fold under high *p*CO_2_ conditions. We also examined photoperiod-length, depth-dependent (0–125 m) responses in rates of ^14^C-PP and ^3^H-Leu incorporation to abrupt *p*CO_2_ increases (to ~750 μatm). In the majority of these depth-resolved experiments (4 of 5 total), rates of ^14^C-PP demonstrated no consistent response to elevated *p*CO_2_. In 2 of 5 depth-resolved experiments, rates of ^3^H-Leu_Dark_ incorporation were lower (10% to 15%) under elevated *p*CO_2_ compared to controls. Our results revealed that rates of ^14^C-PP and bacterial production in this persistently oligotrophic habitat generally demonstrated no or weak responses to abrupt changes in *p*CO_2_. We postulate that any effects caused by changes in *p*CO_2_ may be masked or outweighed by the role that nutrient availability and temperature play in controlling metabolism in this ecosystem.

## Introduction

Human socioeconomic activities, specifically fossil fuel combustion, cement production, and changes in land use, have resulted in progressive increases in atmospheric and oceanic carbon dioxide (CO_2_) inventories [1]. The ocean is a globally important net sink for CO_2_, and as such, increases in atmospheric CO_2_ have raised seawater *p*CO_2_, with concomitant decreases in seawater pH [2–5]. However, studies examining the effects of changes in seawater carbonate chemistry on plankton productivity in open ocean ecosystems are relatively scarce. While an appropriate null hypothesis could be that ocean acidification may lead to no significant changes in microbial contributions to biogeochemical cycling [6], testing such a hypothesis demands rigorous experimental evidence. Previous results and observations suggest that, either as individual species or microbial assemblages, marine microbial physiology may be affected by increases in *p*CO_2_ [7–14]. However, the reported signs and magnitudes of the effects vary [14]. Whether these changes in microbial physiology are large enough to impact ocean biogeochemical cycles remains an important unanswered question.

To date, relatively little is known about the capacity of phytoplankton to adapt or acclimate to changes in the seawater carbonate system, which are likely to have complex influences on ocean biology. Most contemporary lineages of phytoplankton evolved during periods in Earth’s history when atmospheric and oceanic CO_2_ inventories were considerably greater than today [15,16]. Indeed, for many algal species ribulose-1,5-bisphosphate carboxylase/oxygenase (RuBisCO), the enzyme that catalyzes the initial steps of carbon fixation, is less than half saturated at present day *p*CO_2_ [17]. As a consequence, many algae, including cyanobacteria, appear to possess mechanisms for concentrating CO_2_ [18]. Experiments examining the effects of elevated *p*CO_2_ on natural phytoplankton communities have yielded enigmatic results; in several studies, rates of production have increased under elevated *p*CO_2_ [9,14,19], although in other cases, no significant changes in rates of production have been observed [20,21]. There is also compelling evidence that the decline in carbonate ion concentrations that accompanies decreases in seawater pH can be detrimental to the growth of calcifying microorganisms [7], although this appears species-specific [22] and may not apply in the tropical oceans [23]. Additionally, decreases in seawater pH could affect other aspects of seawater chemistry, including altering availability of nitrogen substrates (i.e. decreasing ammonia relative to ammonium) and iron, with concomitant impacts on key processes in the marine nitrogen cycle [13,24].

Numerous laboratory-based *p*CO_2_ manipulation studies have examined the response of specific organisms to changes in seawater carbonate chemistry, where growth conditions are controlled and organisms are generally examined in isolation [12,25]. Other studies have examined natural planktonic communities, including work in the oligotrophic open ocean [8,9,20,21]. Recent mesocosm experiments conducted in nearshore waters found that elevated *p*CO_2_ shifted the partitioning of carbon fixed via photosynthesis from the particulate to the dissolved phase [26] and increased bacterial growth [10]. These intriguing results highlight the need for experiments examining the effects of increased *p*CO_2_ on plankton growth in the open ocean, where a major fraction of global productivity occurs [27] with a significant fraction of the production being partitioned into the dissolved phase which supports the microbial food web [28]. Increasing seawater *p*CO_2_ could impact heterotrophic bacterial growth, through both direct changes to metabolic rates, for example alteration of enzymatic activities [29–31], or indirectly through changes in organic matter production or substrate lability [10,32]. Even small changes in rates of bacterial consumption of organic matter could have a large impact on carbon and nutrient cycling.

In this study, we conducted abrupt perturbations to the seawater carbonate system in the waters of the oligotrophic North Pacific Subtropical Gyre (NPSG) to artificially alter seawater *p*CO_2_ to conditions projected for the surface ocean within the next 50 to 100 years [1]. During these experiments, we examined how such abrupt changes in *p*CO_2_ influenced rates of primary and bacterial production in the near-surface ocean during a series of incubation experiments (2–5 days in duration). We also conducted a series of short-term (~12 hours; photoperiod) depth-resolved experiments to evaluate possible influences of increased *p*CO_2_ on microbial production throughout the euphotic zone (0–125 m). Experiments were conducted in the open ocean of the NPSG, one of the largest biomes on the planet, and hence in an ecosystem that plays a major role in the global cycles of bioelements.

## Materials and methods

### Experimental design

**Fig 1:**
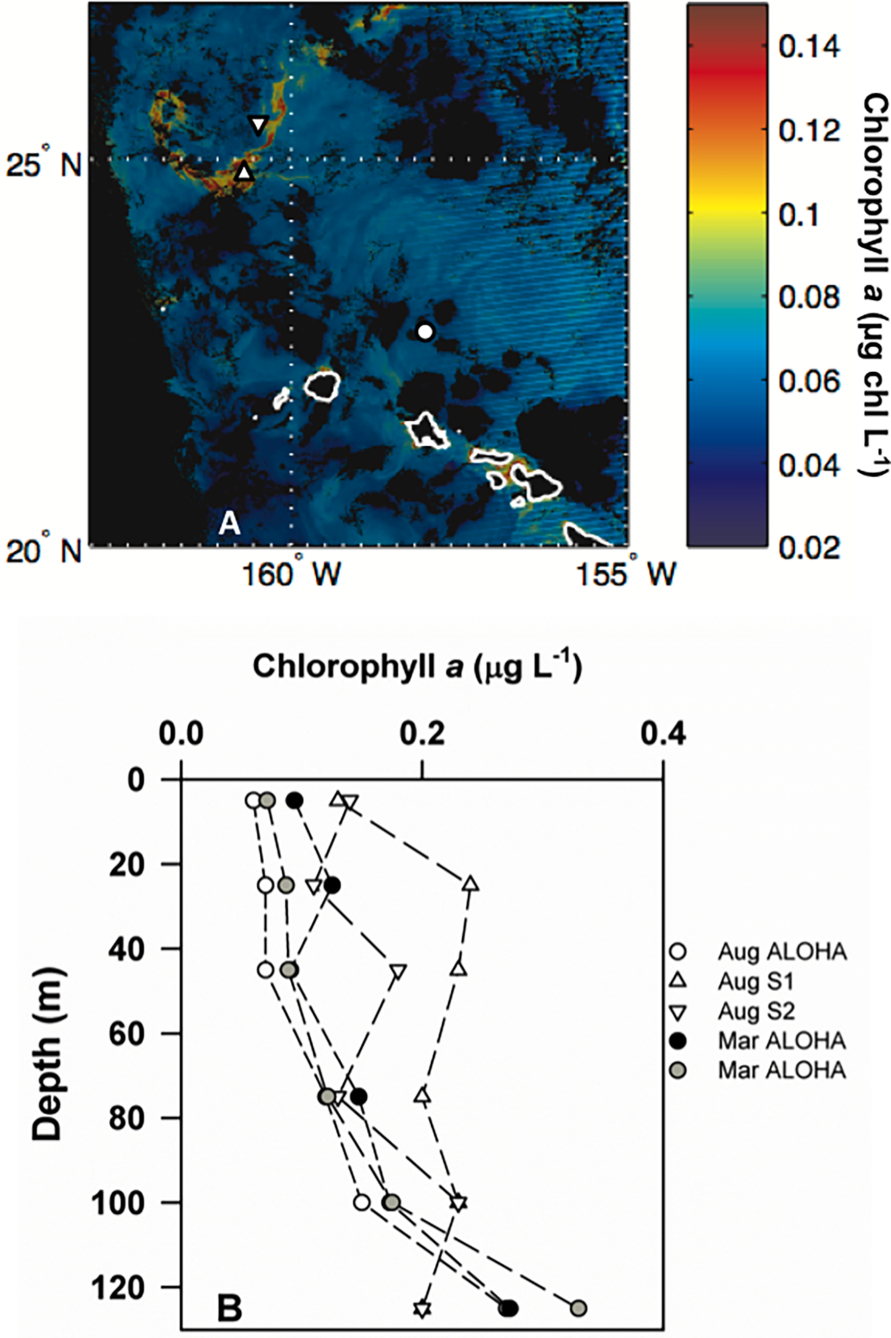
Chlorophyll concentrations and station locations. Satellite ocean color image depicting distributions and concentrations of near-surface ocean chlorophyll *a* (μg/ L^-1^) in the proximity of the Hawaiian Islands on August 21, 2010 (panel A). This image was derived from MODIS Aqua data using the color index (CI) algorithm [38] with no flags applied. The locations of the three stations occupied in August 2010 are also indicated; Station ALOHA is depicted as a circle, while stations S1 and S2 are depicted by triangles. Depth profiles of chlorophyll *a* during the depth-dependent experiments are also shown (panel B).

All seawater carbonate system manipulation experiments were performed on Hawaii Ocean Time-series (HOT) program cruises to Station ALOHA (22° 45’ N 158° W), the field site of the HOT program (between June 2010 and September 2012) or during two process cruises conducted in the vicinity of Station ALOHA (August 2010 and March 2011; Fig 1). Two types of carbonate system manipulation experiments were performed. The first kind of experiments (hereafter “bubbling”) were performed as described in Böttjer et al. [21]. Briefly, near-surface (5–25 m) ocean seawater was collected near midnight using polyvinyl chloride sampling bottles attached to a conductivity-temperature-density (CTD) rosette, and subsampled from CTD rosette bottles under minimal light into acid-washed 20 L polycarbonate carboys fitted with sterile caps with ports for introducing and venting gases. After filling, carboys were placed into shaded (~50% surface irradiance) surface seawater-cooled incubators. Targeted *p*CO_2_ levels were attained by bubbling control carboys with air (~387 μatm *p*CO_2_) and treatment carboys with a mixture of air and CO_2_ (targeting ~750 or ~1100 μatm *p*CO_2_) for 6–8 hours (< 3 L min^-1^). Mixing and delivery of air or mixed air and CO_2_ was regulated by use of mass flow controllers. During the initial 6–8 hours of bubbling, subsamples were collected regularly for measurements of seawater pH (see below for methods), and together with measurements of total alkalinity (TA) were used to estimate seawater *p*CO_2_ using the ‘seacarb’ package [33] in the R statistical environment, with default settings for the carbonate dissociation constants [34–37]. Once the target *p*CO_2_ was reached, the rate of bubbling was reduced (≤ 1.5 L min^-1^) for the duration of the experiment. Subsequent sampling was conducted before dawn at each time point, with the initial sample taken post equilibration considered the beginning of the experiment. Sampling was performed by applying positive pressure to the carboys and subsampling for measurements of TA, dissolved inorganic carbon (DIC), chlorophyll *a*, ^14^C-based primary productivity (^14^C-PP), and rates of ^3^H-leucine (^3^H-Leu) incorporation (as a proxy for bacterial production).

Additional CO_2_ perturbation experiments were conducted at either ambient (~387 μatm) or elevated (~750 μatm) seawater *p*CO_2_ to evaluate depth-dependent responses in ^14^C-PP and ^3^H-Leu incorporation to perturbation of the seawater carbonate system. For these experiments, samples were incubated *in situ* to simulate the vertical gradients in light and temperature representative of the depths from which samples were originally collected and rates of ^14^C-PP and ^3^H-Leu incorporation were measured. Seawater was collected before dawn from six euphotic zone depths (5, 25, 45, 75, 100, and 125 m) and subsampled from the CTD rosette bottles under minimal light into acid-washed 20 L polycarbonate carboys. These carboys were left untreated (controls) or amended with trace metal grade hydrochloric acid (43 mL of 0.1 N HCl) to increase *p*CO_2_ to ~750 μatm (elevated *p*CO_2_ treatments) while minimizing potential changes to TA through additions of sodium bicarbonate (4 mmol). Once the carboys had been amended, seawater from each depth was subsampled into triplicate acid-cleaned 500 mL polycarbonate bottles and acid-cleaned 40 mL polycarbonate centrifuge tubes for subsequent measurements of ^14^C-PP and ^3^H-Leu incorporation, respectively. Following addition of radioactive substrates (see below), these bottles and tubes were affixed to a free-drifting array and incubated *in situ* at the depths of sample collection for the duration of the photoperiod (dawn to dusk).

### Measurements of TA, DIC, and pH

Seawater samples for DIC and TA were collected from each carboy at every time point to evaluate the stability of the carbonate system during bubbling. Samples for determination of carbon system components (TA, DIC, and pH) were collected and analyzed following HOT program protocols [5,39]. DIC and TA samples were collected from carboys into precombusted 300 mL borosilicate bottles. Care was taken to avoid introducing bubbles into the sample during filling, and bottles were allowed to overflow three times during filling. Once filled, each sample was immediately fixed with 100 μL of a saturated solution of mercuric chloride; bottles were capped with a grease seal, and stored in the dark for later analysis. DIC concentrations were determined coulometrically using a Versatile INstrument for the Determination of Total inorganic carbon and Titration Alkalinity 3S (VINDTA) system [40]. TA was determined using an automated, closed-cell potentiometric titration. The precision and accuracy of these measurements were validated by comparison to a certified seawater CO_2_ reference sample [40], with accuracies of approximately ±3 μmol L^-1^ for TA and ±1 μmol L^-1^ for DIC. Seawater pH (measured at 25°C) was analyzed using spectrophotometric detection of m-cresol purple with a precision of 0.001 [5,41].

### Measurements of ^14^C-PP

Rates of ^14^C-PP were measured at each sampling time point during the bubbling experiments. At each pre-dawn sampling, seawater was subsampled from the carboys into acid cleaned 500 mL polycarbonate bottles, and each bottle was amended with ~1.85 MBq ^14^C-bicarbonate. The total radioactivity added to each sample bottle was determined post-incubation by subsampling 250 μL aliquots of seawater into scintillation vials containing 500 μL of β-phenylethylamine. Bottles were placed in shaded (~50% irradiance) surface seawater-cooled incubators for the duration of the photoperiod. After sunset, 100 mL from each sample bottle was filtered at low vacuum (<50 mm Hg) onto 25 mm diameter, 0.2 μm porosity polycarbonate membrane filters. The filters were then stored frozen in 20 mL scintillation vials until analysis at the shore-based laboratory. At the shore-based laboratory, filters were acidified by the addition of 1 mL of 2 N hydrochloric acid, and allowed to passively vent for at least 24 hours in a fume hood to remove all inorganic ^14^C, followed by addition of 10 mL Ultima Gold LLT liquid scintillation cocktail. The resulting radioactivity was determined on a Perkin Elmer 2600 liquid scintillation counter.

Measurements of ^14^C-PP from the depth-dependent experiments were conducted similarly, except that the samples were incubated *in situ* at the respective depths of collection on a free-drifting array for the duration of the photoperiod, and the incubations were terminated via sequential size fractionated filtration onto 10 μm, 2 μm, and 0.2 μm pore sized polycarbonate filters (25 mm diameter). Filters were treated as previously described for subsequent determination of ^14^C activity.

### ^3^H-leucine incorporation measurements

We measured ^3^H-Leu incorporation into plankton protein as a proxy measurement for bacterial production [42, 43]. Rates of ^3^H-Leu incorporation were measured following incubations conducted in both the light (^3^H-Leu_Light_) and in the dark (through use of black cloth bags; ^3^H-Leu_Dark_ [44]). From the bubbling experiments, 125 mL polyethylene amber bottles were subsampled from each carboy in the pre-dawn hours; 6 aliquots of 1.5 mL were then subsampled from each bottle into 2 mL microcentrifuge tubes (Axygen) containing 20 nmol L^-1 3^H-leucine (final concentration). In addition, 1.5 mL of seawater was subsampled into a 2 mL microcentrifuge tube containing 20 nmol L^-1 3^H-leucine (final concentration) and 100 μL of 100% (w/v) trichloroacetic acid (TCA); these samples served as time zero “blanks”. Samples were incubated for 2 to 12 hours in the same surface seawater-cooled incubator described previously. To terminate incubations, 100 μL of 100% TCA was added to each microcentrifuge tube and tubes were frozen (-20°C) for later processing.

For those experiments incubated *in situ* on the free-drifting array, water was subsampled from each of the control and treatment carboys into 40 mL polycarbonate centrifuge tubes and each tube was inoculated with ^3^H-leucine to a final concentration of 20 nmol L^-1^. Time zero blanks were immediately subsampled from each tube; for these samples, 1.5 mL of seawater was aliquoted into 2 mL microcentrifuge tubes containing 100 μL of 100% TCA. The 40 mL tubes were then affixed to the same free drifting array utilized for the ^14^C-bicarbonate assimilation measurements and samples were incubated under ambient light and in the dark (by placing the tubes in a darkened cloth bag). The array was then deployed for the duration of the photoperiod. After sunset, the array was recovered, and triplicate 1.5 mL subsamples were removed from each of the polycarbonate tubes and aliquoted into 2 mL microcentrifuge tubes containing 100 μL of 100% TCA. The microcentrifuge tubes were frozen (-20°C) for later processing, following the procedures described in Smith and Azam [45].

### Contextual biogeochemical data, statistics, and data analysis

Seawater samples for contextual biogeochemical analyses were collected and analyzed according to HOT program protocols (http://hahana.soest.hawaii.edu/hot/methods/results.html). Measurements of fluorometric chlorophyll *a* concentrations were performed as in Letelier et al. [46]. Samples for analysis of nutrient concentrations were subsampled from the CTD rosette bottles into acid washed polyethylene bottles (125 or 500 mL) and stored upright at -20°C. Combined concentrations of nitrate and nitrite (N+N) were analyzed using the high sensitivity chemiluminescent technique [47,48], while concentrations of soluble reactive phosphorus (SRP) were determined via the magnesium-induced co-precipitation (MAGIC) method [49].

Statistical analyses were performed using Matlab (Mathworks). Data that were not normally distributed were log_10_ transformed prior to subsequent analyses. Statistical differences between rates of ^14^C-PP and ^3^H-Leu incorporation at different *p*CO_2_ levels during our bubbling experiments were determined by two-way analysis of variance (ANOVA), where *p*CO_2_ and time were the factors of variation. For the depth-resolved experiments, we assessed significance for individual depths and for the depth-integrated rates based on the mean and standard deviation of the rates of ^14^C-PP and ^3^H-Leu incorporation in the treatments and controls using Student’s t-tests.

## Results

We conducted a total of 10 shipboard *p*CO_2_ manipulation experiments where seawater *p*CO_2_ was altered by bubbling with CO_2_-air gas mixtures. We measured rates of ^14^C-PP in all 10 of these bubbling experiments, and rates of ^3^H-Leu incorporation were measured in 8 of these experiments. An additional 5 experiments were conducted to evaluate depth-dependent responses in rates of ^14^C-PP and ^3^H-Leu incorporation to elevated *p*CO_2_. Experiments were conducted in all four seasons, spanning the range of conditions typically observed at Station ALOHA (Table 1). With two exceptions, all experiments were conducted with seawater collected at Station ALOHA; two of the depth-resolved experiments (August 26, 2010 and August 28, 2010) were conducted at sampling sites to the northwest of Station ALOHA (termed S1: 24° 45’ N 160° 45’ W and S2: 25° 35’ N 160° 32’ W) where concentrations of chlorophyll *a* in near-surface waters were elevated relative to Station ALOHA (Table 1; Fig 1).

**Table 1:** Starting conditions for experiments.

Month	Year	Station	T (^o^C)	Chlorophyll *a* (μg L^-1^)	^14^C-PP (μmol C L^-1^ d^-1^)	N+N (nmol L^1^)	SRP (nmol L^1^)	DIC (μmol L^-1^)	TA (μmol L^-1^)	pH	*p*CO_2_ (μatm)
June	2010	ALOHA	24.7	0.06	0.26	2	62	2018	2336	8.069	376
August	2010	ALOHA	25.5	0.05	0.29	3	105	1997	2326	8.079	364
August	2010	S1	26.1	0.13	0.63	ND	ND	2041	2357	8.039	419
August	2010	S2	26.0	0.14	0.93	ND	ND	2009	2330	8.052	400
September	2010	ALOHA	25.6	0.07	ND	4	86	1998	2323	8.069	373
October	2010	ALOHA	26.0	0.08	0.42	3	65	1997	2323	8.065	377
January	2011	ALOHA	23.7	0.05	0.21	8	50	2012	2333	8.089	355
March	2011	ALOHA	24.4	0.07	0.43	3	68	1997	2324	8.092	351
April	2011	ALOHA	24.0	0.06	0.17	5	103	1993	2312	8.089	353
September	2012	ALOHA	25.5	0.06	0.32	8	123	1999	2306	8.048	377

Near-surface ocean (5 m) temperatures (T), concentrations of chlorophyll *a*, rates of primary productivity (^14^C-PP), nutrient concentrations (nitrate plus nitrite = N+N and soluble reactive phosphorus = SRP), and carbonate system properties (dissolved inorganic carbon = DIC, total alkalinity = TA, partial pressure of carbon dioxide = *p*CO_2_) during those months when experiments were conducted for this study. ND = no data.

**Fig 2:**
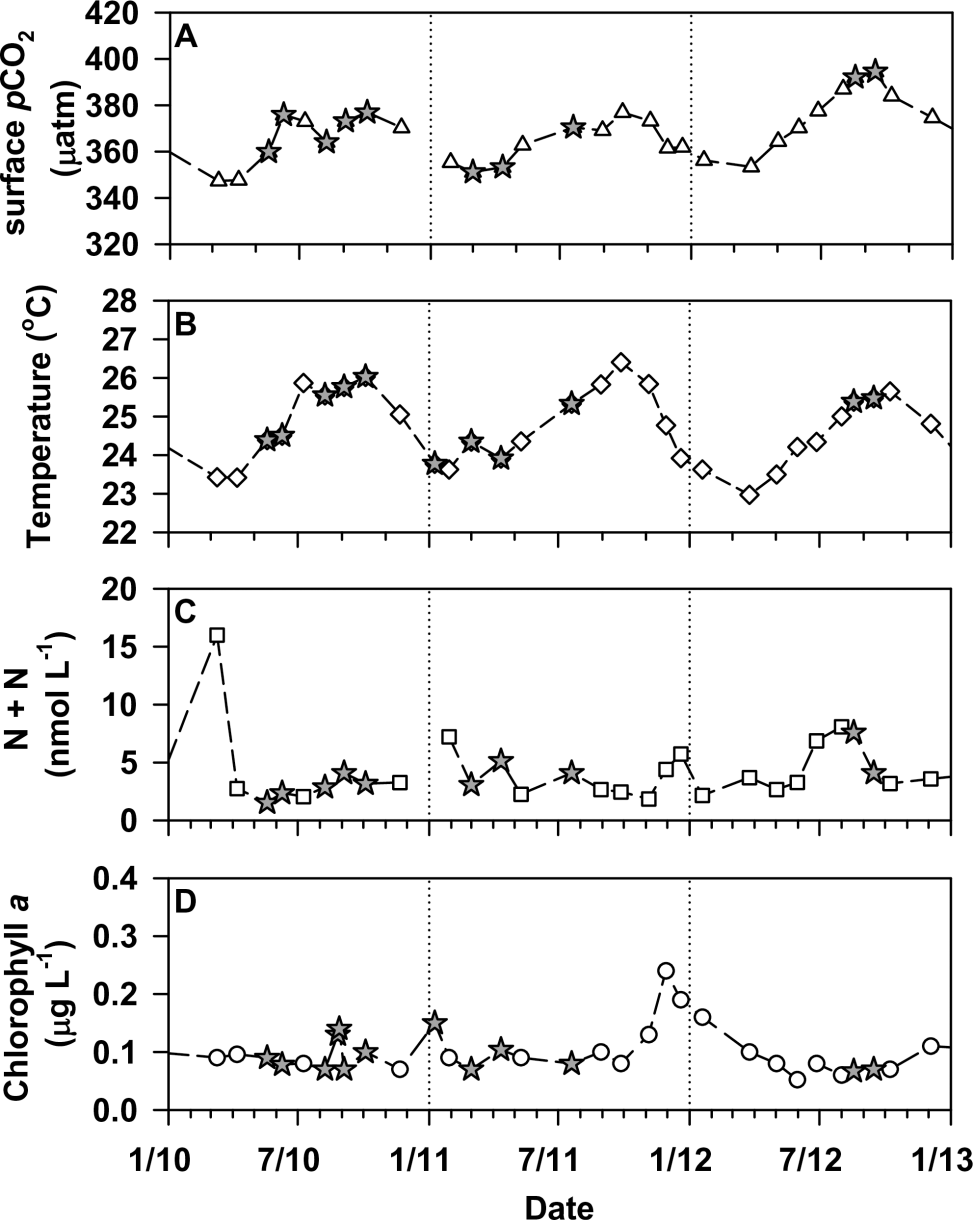
Initial experimental conditions. Mixed layer seawater *p*CO_2_ measured during the period of this study (2010–2012) at Station ALOHA (panel A; triangles). Also shown are temperature at Station ALOHA (panel B; diamonds), concentrations of nitrate + nitrite (N+N; panel C; squares) and chlorophyll *a* (panel D; circles) in near-surface waters (5 m). Grey stars are used to indicate those cruises when bubbling experiments were conducted.

For all bubbling experiments, initial concentrations of N+N and SRP were consistently below 10 nmol L^-1^ and 150 nmol L^-1^, respectively, consistent with HOT program measurements of these nutrients (Table 1; Fig 2). Rates of particulate ^14^C-PP at the beginning of the experiments ranged between 0.17 to 0.93 μmol C L^-1^ d^-1^, with the higher rates measured at those stations to the northwest of ALOHA where chlorophyll *a* concentrations were elevated (Table 1). Seawater *p*CO_2_ in the near-surface waters at the time the experiments were conducted ranged between 351 μatm to 419 μatm, consistent with HOT program observations at Station ALOHA [5,21], while sea surface temperatures ranged between ~24 and 26°C (Table 1).

### Concentrations of chlorophyll *a* and rates of ^14^C-PP and ^3^H-Leu incorporation under elevated *p*CO_2_

**Fig 3:**
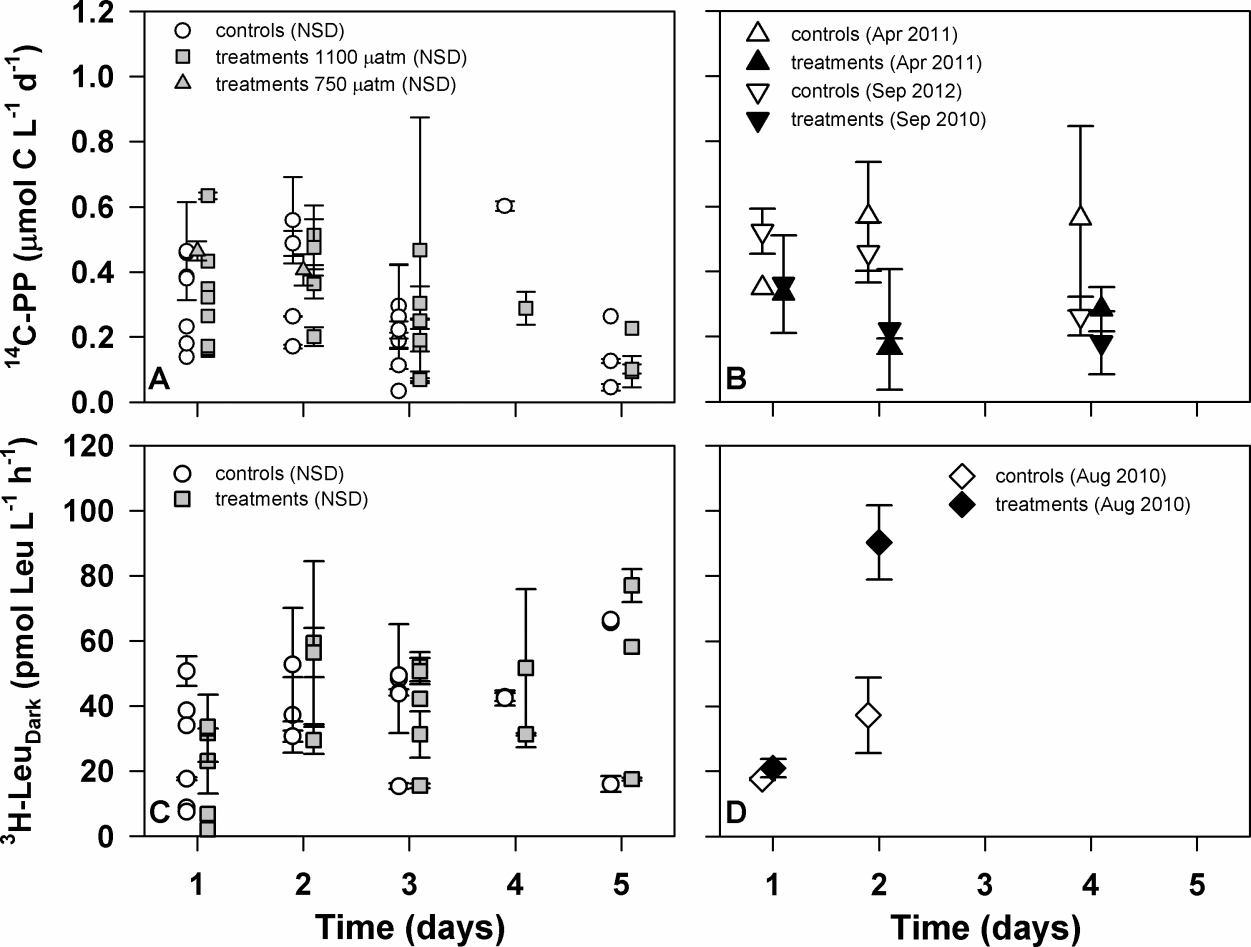
Results of *p*CO_2_ bubbling experiments. Measured rates of ^14^C-PP (panel A) and ^3^H-Leu_Dark_ incorporation (panel C) from all experiments (n = 8 and n = 7, respectively) where no significant differences (NSD) were observed between controls (white circles) and elevated *p*CO_2_ treatments at 1100 μatm (grey squares) and 750 μatm (grey triangles) are depicted. Also shown are rates of ^14^C-PP (panel B) and ^3^H-Leu_Dark_ incorporation (panel D) from experiments where significant differences (two-way ANOVA; p<0.05) were observed between controls (open symbols) and elevated *p*CO_2_ (black symbols) treatments. Dates of experiments showing significant differences are given in the legend.

In 8 out of 10 bubbling experiments rates of ^14^C-PP in the elevated *p*CO_2_ treatments were not significantly different than rates measured in the controls (two-way ANOVA; p>0.05; Table 2; Fig 3). In the remaining 2 bubbling experiments (April 2011 and September 2012) rates of ^14^C-PP in the controls were significantly greater than rates measured in the enhanced *p*CO_2_ treatments (two-way ANOVA, p<0.05; Table 2); notably, concentrations of N+N and SRP in both of these experiments were elevated relative to other experiments (Table 1). There were no significant interactions between *p*CO_2_ and time for any of the experiments (two-way ANOVA; p>0.05). The median value of the percent differences between treatments and controls ([treatments—controls] / controls) across all time points was -6% (mean 3%, standard deviation 51%; Table 2), with the treatments differing from the controls during 20% of the sampling occasions.

**Table 2:** Percent difference between controls and elevated *p*CO_2_ treatments.

Start date	days	^14^C-PP (% difference)	p value	Chlorophyll *a* (% difference)	p value	^3^H-Leu_Dark_ incorporation (% difference)	p value	^3^H-Leu_Light_ incorporation (% difference)	p value
Aug. 6, 2010	1	14		4		ND		ND	
	2	94 ±34		1 ±0		ND		ND	
	3	58 ±147	NSD	-8 ±1	NSD	ND		ND	
Aug. 21, 2010	1	0 ±33		7 ±0		19 ±16		-4 ±10	
(750 μatm)	2	-27 ±26	NSD	-10 ±1	NSD	143 ±62	<0.005	130 ±92	NSD
Aug. 21, 2010	1	37 ±35		-1 ±1		31 ±57		19 ±52	
	2	-35 ±27	NSD	-16 ±1	NSD	60 ±76	NSD	58 ±77	NSD
Aug. 23, 2010	1	10.2		6		315		345	
	2	-52 ±46		12 ±2		-4 ±15		-7 ±12	
	3	ND	NSD	ND	NSD	-35 ±39	NSD	-30 ±17	NSD
Sep. 3, 2010	1	14		27		ND		ND	
	2	18 ±17		10 ±1		ND		ND	
	3	-7 ±45	NSD	6 ±1	<0.05	ND		ND	
Oct. 3, 2010	1	-57		-11		-77		ND	
	3	16 ±65		2 ±1		18 ±10		ND	
	4	-52 ±9	NSD	-13 ±2	NSD	21±57	NSD	ND	
Jan. 11, 2011	1	-30		-6		-14		11	
	3	-14		7		-3		27	
	5	121	NA	17	NA	-11	NA	-16	NA
Mar. 3, 2011	1	-7		13		-1		-26	
	3	-14 ±20		-18 ±2		3 ±8		2 ±8	
	4	-26 ±38	NSD	-22 ±2	NSD	16 ±8	NSD	-4 ±5	NSD
Mar. 17, 2011	1	-4		-11		-9		1	
	3	102 ±13		-23 ±1		1 ±7		-26 ±14	
	5	123 ±48	NSD	4 ±1	NSD	9 ±16	NSD	11 ±5	NSD
Apr. 15, 2011	1	-5		-50		ND		ND	
	2	-70 ±36		-63 ±1		ND		ND	
	3	-50 ±58	<0.05	-43 ±3	<0.0005	ND		ND	
Sep. 6, 2012	1	-31 ±32		-29 ±3		-35 ±23		-36 ±26	
	2	-52 ±46		-39 ±3		7 ±36		3 ±44	
	4	-31 ±44	<0.05	-23 ±2	<0.005	-26 ±6	NSD	-31 ±12	NSD

Percent differences ([treatments—controls] / controls) between *p*CO_2_ elevated treatments (~1100 μatm except August 21, 2010) and controls (ambient, ~387 μatm) in bubbling experiments for ^14^C-PP, chlorophyll *a*, ^3^H-Leu_Dark_ incorporation, and ^3^H-Leu_Light_ incorporation. Differences between controls and treatments for each full experiment are reported as p-values (two-way ANOVA). NSD = not significantly different (p>0.05). ND = no data. NA = statistical test not applicable, due to lack of replication.

In addition, during most experiments (7 of 10) concentrations of chlorophyll *a* in the elevated *p*CO_2_ treatments were not significantly different than in the controls (two-way ANOVA; p>0.05; Table 2). In a single experiment (September 2010), chlorophyll *a* concentrations in the elevated *p*CO_2_ treatments were greater than those in the controls. In contrast, rates of ^14^C-PP and concentrations of chlorophyll *a* were greater in controls relative to the elevated *p*CO_2_ treatments in two of the experiments (April 2011 and September 2012; two-way ANOVA; p<0.05; Table 2; Fig 3). We also normalized our measured rates of ^14^C-PP to concentrations of chlorophyll *a*, and in 9 of 10 experiments there was no significant difference between controls and elevated *p*CO_2_ treatments (two-way ANOVA; p>0.05; S1 Fig). In the experiment conducted in March 2011, chlorophyll *a* normalized rates of ^14^C-PP were greater in elevated *p*CO_2_ treatments than in controls (two-way ANOVA; p<0.05; S1 Fig).

We also examined possible responses in rates of ^3^H-Leu incorporation during the seawater carbonate system manipulation experiments (Table 2). In total, rates of ^3^H-Leu_Dark_ incorporation were determined in 7 of the bubbling experiments, with coincident measurements of rates of ^3^H-Leu_Light_ incorporation in 6 of these 7 experiments (Table 2). In the enhanced *p*CO_2_ treatments rates of ^3^H-Leu_Dark_ incorporation were similar to those measured in the controls, ranging between 7 and 41 pmol Leu L^-1^ h^-1^, with measurements at subsequent time points ranging from 4 to 98 pmol Leu L^-1^ h^-1^ (Fig 3). Rates of ^3^H-Leu_Light_ incorporation in the controls ranged between 9 and 61 pmol Leu L^-1^ h^-1^ at the beginning of the experiments, and between 21 and 84 pmol Leu L^-1^ h^-1^ at subsequent time points. In the enhanced *p*CO_2_ treatments, rates of ^3^H-Leu_Light_ incorporation at the beginning of the experiments ranged from 15 to 65 pmol Leu L^-1^ h^-1^, and from 17 to 99 pmol Leu L^-1^ h^-1^ at subsequent time points. In 5 out of the 7 experiments where rates were measured, ^3^H-Leu_Dark_ incorporation rates increased significantly over time in both the controls and treatments (two-way ANOVA, p<0.05; Fig 3). Similarly, in 4 of the 6 experiments in which ^3^H-Leu_Light_ incorporation was measured rates increased significantly over the duration of the experiment in both the controls and treatments (two-way ANOVA, p<0.05). However, in the majority of experiments there were no significant differences in the enhanced *p*CO_2_ treatments relative to the controls (two-way ANOVA, p>0.05; Table 2). In a single experiment (August 2010) rates of ^3^H-Leu_Dark_ incorporation in the *p*CO_2_ treatments (750 μatm) were significantly greater than the controls (two-way ANOVA, p<0.05; Table 2; Fig 3). The median values of the percent differences ([CO_2_ treatments—ambient controls]/controls) in rates of ^3^H-Leu_Dark_ and ^3^H-Leu_Light_ incorporation across all time points were 2% (mean 19%, standard deviation 78%) and 1% (mean 22%, standard deviation 87%) respectively (Table 2). The resulting differences (%) were significantly different from zero in less than 20% of experimental time points (13% and 15% for ^3^H-Leu_Dark_ and ^3^H-Leu_Light_ incorporation, respectively).

### Depth-dependent responses in ^14^C-PP and ^3^H-Leu incorporation to elevated *p*CO_2_

In addition to conducting *p*CO_2_ perturbation experiments where near-surface ocean water was bubbled continuously for up to 5 days, we also conducted 5 experiments where we examined short-term, daytime (dawn to dusk), depth-dependent responses in rates of ^14^C-PP and ^3^H-Leu incorporation to perturbations in seawater *p*CO_2_. For these experiments, seawater *p*CO_2_ at 6 discrete depths in the upper ocean was perturbed through the addition of acid (and bicarbonate to maintain constant alkalinity) and incubated *in situ* on a free-drifting array. For samples in the upper euphotic zone (<45 m), the *p*CO_2_ derived from measurements of DIC and TA was within ~20% of the target *p*CO_2_ (750 μatm), while in the lower euphotic zone (>75 m) the derived *p*CO_2_ values were uniformly greater (by 2–52%) than the target *p*CO_2_ (S2 Fig). This was likely due to a combination of depth-dependent natural increases in *p*CO_2_ and the greater variability of the seawater carbonate system in the lower euphotic zone compared to surface waters [50].

**Fig 4:**
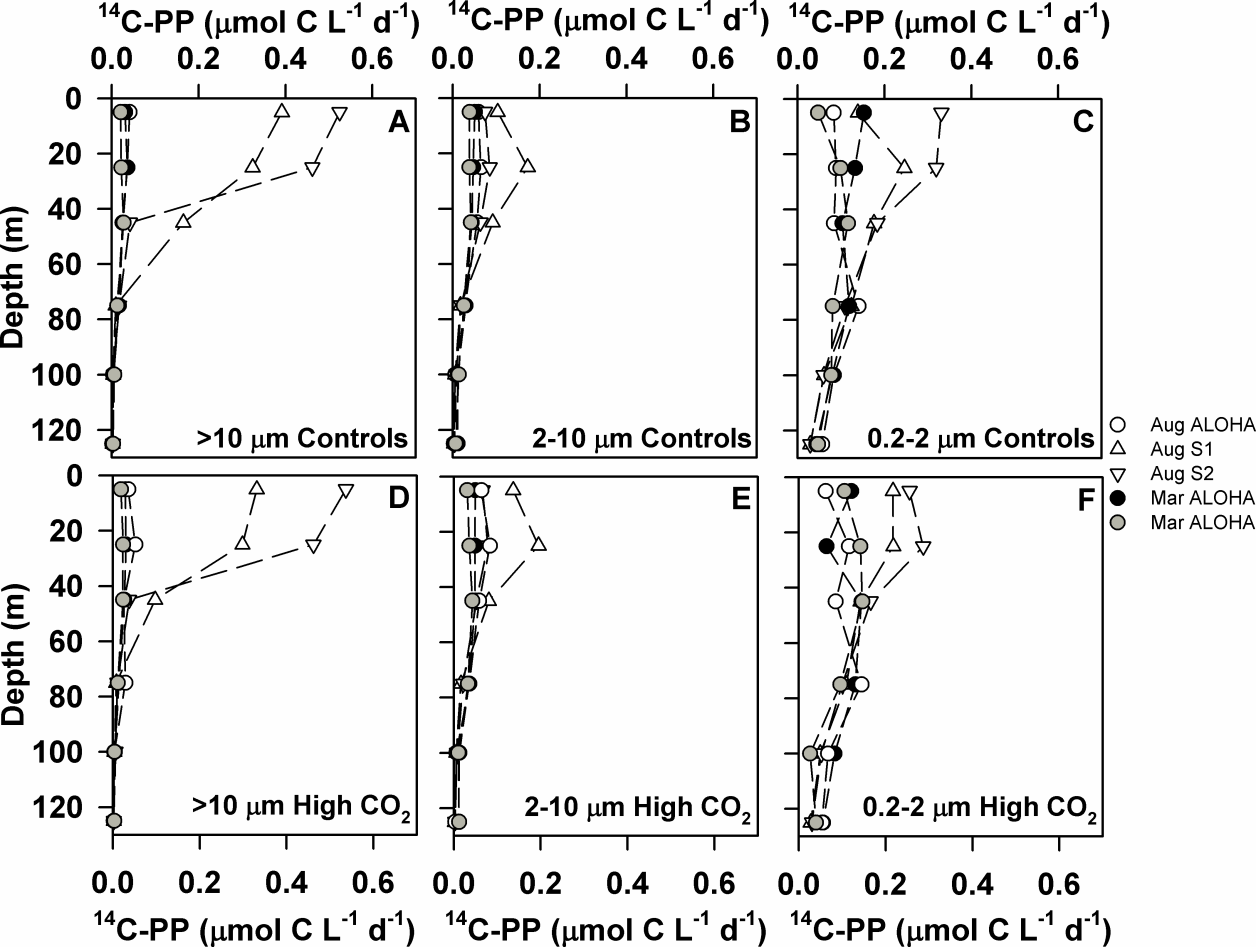
Depth-resolved measurements of size fractionated ^14^C-PP during cruises in August 2010 and March 2011. Rates of ^14^C-PP in the >10 μm size fraction (panels A and D), 2–10 μm size fraction (panels B and E), and 0.2–2 μm size fraction (panels C and F) under ambient (387 μatm; panels A-C) and elevated *p*CO_2_ (750 μatm) conditions (panels D-F).

Rates of ^14^C-PP were measured from size fractionated water samples (>10 μm, 2–10 μm, and 0.2–2 μm) from all six depths from both the controls and *p*CO_2_-perturbed treatments (Table 3; Fig 4). Overall, rates of ^14^C-PP in all of the size fractions were greatest at stations S1 and S2, where concentrations of chlorophyll *a* were also elevated (Fig 1). Rates of ^14^C-PP in the >10 μm size fraction at these two stations ranged from 0.4 to 0.5 μmol C L^-1^ d^-1^, approximately an order of magnitude greater than rates observed at ALOHA (Fig 4). The resulting depth-integrated upper euphotic zone (0–45 m) rates of ^14^C-PP in the >10 μm size fraction ranged between 1.0 and 17.7 mmol C m^-2^ d^-1^, with average rates at S1 and S2 (August 26 and 28, 2010) ~11-fold greater than at ALOHA (Table 3). Similarly, rates measured at S1 and S2 were elevated in the 2–10 μm and 0.2–2 μm size fractions, with depth-integrated (0–45 m) rates at these stations ranging from 3.1 to 6.8 and 8.8 to 13.2 mmol C m^-2^ d^-1^, respectively (Table 3) compared to 1.6 to 3.2 mmol C m^-2^ d^-1^ and 3.8 to 6.0 mmol C m^-2^ d^-1^, respectively at Station ALOHA. In the lower euphotic zone (75–125 m), rates of ^14^C-PP in the two larger size fractions at Station ALOHA were 2- to 5- fold lower than in the upper euphotic zone, with rates in the 0.2–2 μm size fraction in the lower euphotic zone as much as 2.5-fold lower than the upper ocean (Table 3).

**Table 3:** Depth-integrated rates of ^14^C-PP.

Date treatment	^14^C-PP >10 μm (mmol C m^-2^ d^-1^)		^14^C-PP >10 μm (mmol C m^-2^ d^-1^)		^14^C-PP >10 μm (mmol C m^-2^ d^-1^)		^14^C-PP 2–10 μm (mmol C m^-2^ d^-1^)		^14^C-PP 2–10 μm (mmol C m^-2^ d^-1^)		^14^C-PP 2–10 μm (mmol C m^-2^ d^-1^)		^14^C-PP 0.2–2 μm (mmol C m^-2^ d^-1^)		^14^C-PP 0.2–2 μm (mmol C m^-2^ d^-1^)		^14^C-PP 0.2–2 μm (mmol C m^-2^ d^-1^)	
	0–45		75–125		0–125		0–45		75–125		0–125		0–45		75–125		0–125	
Aug. 21, 2010																		
390 μatm	1.6±0.1	A	0.3±0.0	C	2.5±0.1	ns	2.6±0.2	ns	0.5±0.0	B	4.6±0.2	A	3.9±0.7	ns	4.5±0.1	A	11.8±1.0	ns
750 μatm	1.8±0.1		0.5±0.0		3.1±0.6		3.2±0.3		0.6±0.0		5.3±0.3		4.1±0.8		4.2±0.2		11.7±0.8	
Aug. 26, 2010 (S1)																		
390 μatm	14.0±1.0	A	0.2±0.0	ns	16.8±1.0	A	5.9±0.3	ns	0.4±0.0	ns	8.0±0.3	ns	8.8±1.0	ns	3.4±0.3	ns	16.7±1.0	ns
750 μatm	11.9±0.7		0.2±0.0		13.7±0.7		6.8±1.0		0.4±0.0		8.6±1.0		9.0±1.1		3.0±0.2		15.8±1.4	
Aug. 28, 2010 (S2)																		
390 μatm	17.5±2.3	ns	0.4±0.5	ns	18.8±2.3	ns	3.5±0.5	ns	0.5±0.1	ns	5.2±0.5	ns	13.2±06	A	3.2±0.1	ns	20.8±0.6	A
750 μatm	17.7±1.5		0.3±0.1		18.7±1.5		3.1±0.1		0.5±0.0		4.7±0.2		11.2±0.8		2.9±0.2		18.1±0.9	
Mar. 14, 2011																		
390 μatm	1.4±0.4	ns	0.3±0.0	ns	2.3±0.4	ns	2.1±0.2	ns	0.8±0.1	ns	4.1±0.2	ns	6.0±1.3	ns	4.0±0.3	ns	13.3±1.3	ns
750 μatm	1.3±0.2		0.3±0.0		2.2±0.2		2.2±0.3		0.9±0.1		4.4±0.3		4.5±0.6		4.4±0.3		13.1±1.2	
Mar. 16, 2011																		
390 μatm	1.0±0.2	ns	0.3±0.0	ns	2.0±0.2	ns	1.8±0.2	ns	0.7±0.1	ns	3.5±0.3	ns	3.8±1.9	ns	3.5±1.3	ns	10.3±2.4	ns
750 μatm	1.0±0.2		0.3±0.0		1.8±0.2		1.6±0.1		0.8±0.1		3.6±0.3		5.9±1.0		2.4±0.6		11.9±1.6	

Depth-integrated rates of ^14^C-PP measured under both ambient (387 μatm) and enhanced *p*CO_2_ (750 μatm) conditions in both upper (0–45 m) and lower euphotic zones (75–125 m) for three plankton size fractions. Significant differences between rates in controls and treatments are indicated by capital letters (two-sample t-Test), lack of significant difference indicated by “ns”.

A: p<0.05.

B: p<0.01.

C: p<0.005.

**Fig 5:**
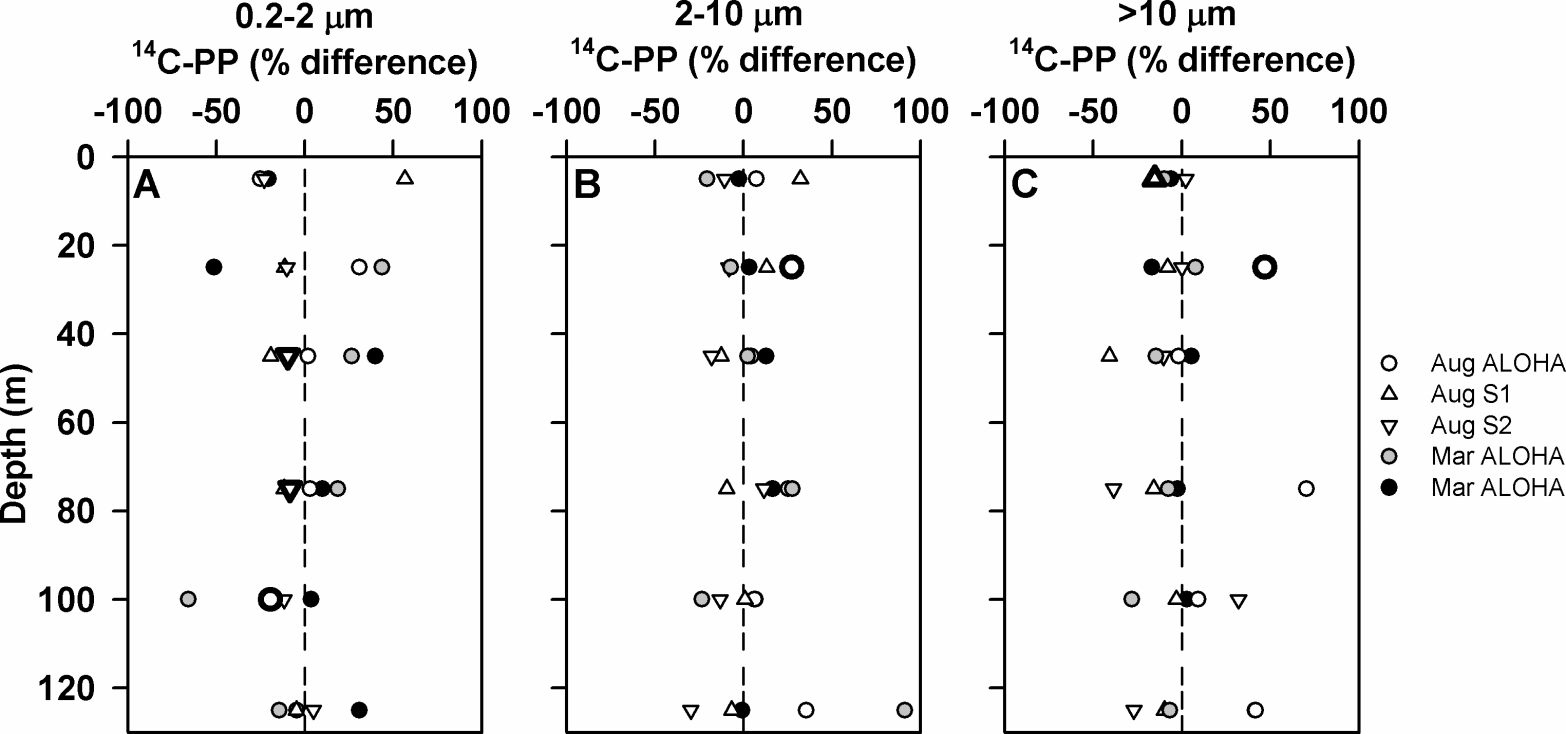
Percent differences in ^14^C-PP between controls and elevated *p*CO_2_ treatments. Shown are percent differences ([treatments (750 μatm) - controls (387 μatm)] / controls) for rates of ^14^C-PP from depth-resolved experiments during cruises in August 2010 and March 2011 for >10 μm size class (panel A), 2–10 μm size class (panel B), and the 0.2–2 μm size class (panel C). Dashed line indicates zero. Bold symbols indicate significant differences between controls and treatments.

Consistent with results from the bubbling experiments, overall abrupt increases in *p*CO_2_ had little or no effect on rates of ^14^C-PP in these depth-resolved experiments. For at least one of the depths examined in 3 of the depth-resolved experiments, rates of ^14^C-PP in the controls were greater than the *p*CO_2_-elevated treatments (t-Test; p<0.05; Fig 5). However, in 1 of the 5 experiments (occurring in August 2010) rates of ^14^C-PP in the *p*CO_2_-elevated treatments were greater than in the controls for both the >10 μm and 2–10 μm size fractions at a single depth (25 m; t-Test; p<0.005 and p<0.05, respectively; Fig 5). There were no consistent differences in depth-integrated rates of ^14^C-PP in the upper euphotic zone between controls and elevated *p*CO_2_ treatments (0–45 m; Table 3; t-Test; p>0.05). In 4 of the 5 depth-resolved experiments there were no consistent differences in rates of ^14^C-PP between controls and *p*CO_2_ elevated treatments in the lower euphotic zone. In one of the experiments (August 2010) conducted at Station ALOHA, rates of ^14^C-PP in the lower euphotic zone (75–125 m) were significantly greater in the >10 μm and 2–10 μm size fractions in the *p*CO_2_ perturbed treatments relative to the controls (t-Test; p<0.05; Table 3).

**Fig 6:**
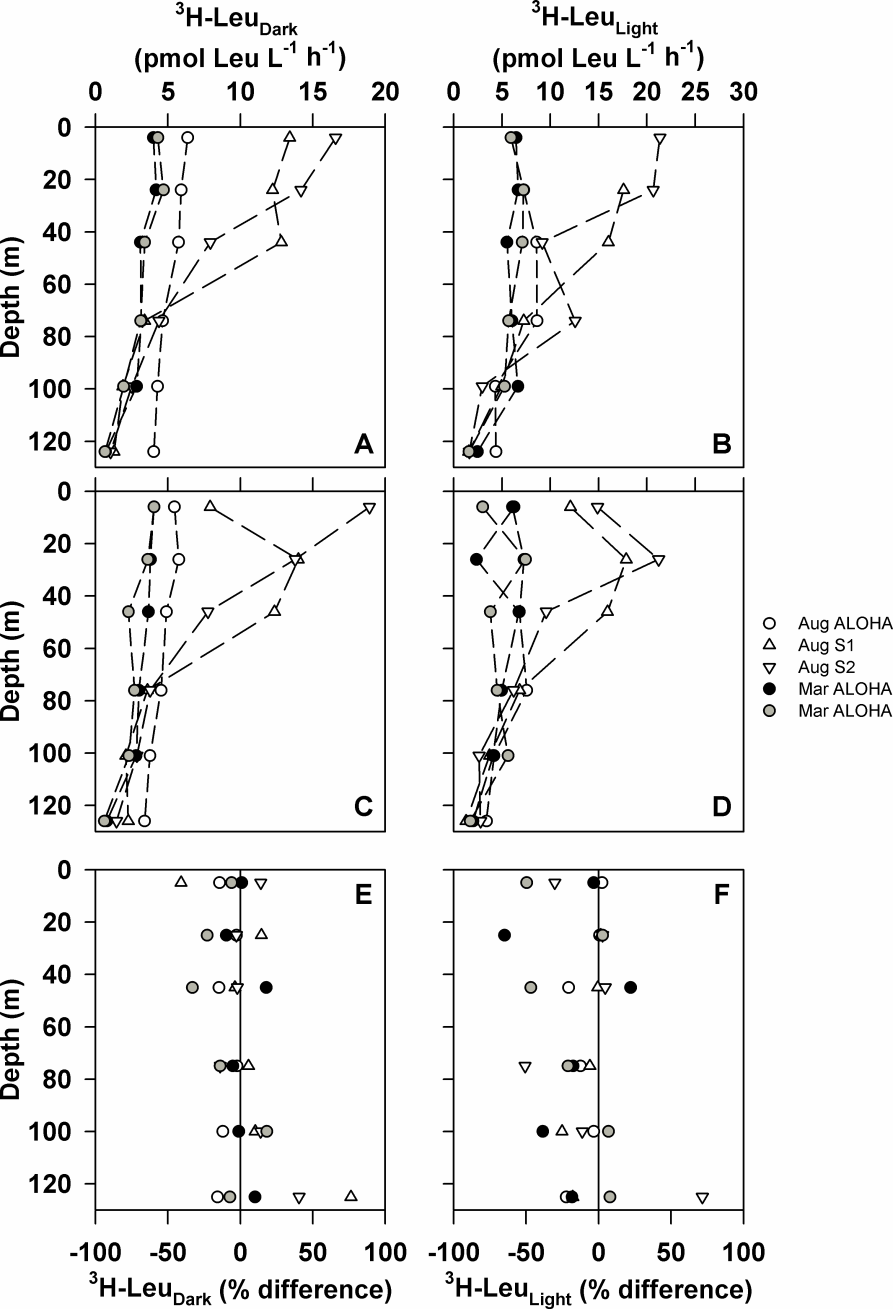
Depth-resolved rates of ^3^H-Leu incorporation in August 2010 and March 2011. Rates of ^3^H-Leu incorporation (pmol Leu L^-1^ h^-1^) in the dark for both ambient and elevated seawater *p*CO_2_ (~390 and 750 μatm, respectively) are shown (panels A and C, respectively), as are rates in the light for both ambient and elevated *p*CO_2_ (panels B and D, respectively). Also shown are percent differences between treatments ([treatments—controls] / controls) for ^3^H-Leu_Dark_ incorporation (panel E) and ^3^H-Leu_Light_ incorporation (panel F).

Similar to rates of ^14^C-PP, rates of ^3^H-Leu_Dark_ and ^3^H-Leu_Light_ incorporation were greater at stations S1 and S2 than at ALOHA (one-way ANOVA; p<0.0001 for both; Fig 6). The resulting depth-integrated (0–125 m) rates of ^3^H-Leu_Dark_ and ^3^H-Leu_Light_ incorporation were significantly greater in controls than in elevated *p*CO_2_ treatments in 2 of 5 and 5 of 5 depth-resolved experiments, respectively (t-Test; p<0.05; Table 4). In the upper euphotic zone (0–45 m), rates of ^3^H-Leu_Dark_ and ^3^H-Leu_Light_ incorporation were significantly lower in the elevated *p*CO_2_ treatments than in the controls in 2 of 5, and 3 of 5 experiments, respectively (t-Test; p<0.05; Table 4). In 4 of the 5 experiments, rates of ^3^H-Leu_Light_ incorporation in the lower euphotic zone (75–125 m) were significantly lower in the elevated *p*CO_2_ treatments than the controls (t-Test; p<0.05; Table 4).

**Table 4:** Depth-integrated rates of ^3^H-Leu incorporation.

Date treatment	^3^H-Leu_Dark_ (nmol Leu m^-2^ h^-1^)		^3^H-Leu_Dark_ (nmol Leu m^-2^ h^-1^)		^3^H-Leu_Dark_ (nmol Leu m^-2^ h^-1^)		^3^H-Leu_Light_ (nmol Leu m^-2^ h^-1^)		^3^H-Leu_Light_ (nmol Leu m^-2^ h^-1^)		^3^H-Leu_Light_ (nmol Leu m^-2^ h^-1^)	
	0–45 m		75–125 m		0–125 m		0–45 m		75–125 m		0–125 m	
Aug. 21, 2010												
390 μatm	272±9	A	216 ±6	ns	643 ±11	B	321±12	ns	271±10	A	849 ±16	C
750 μatm	246±9		193±11		581±14		306±7		242±8		763 ±13	
Aug. 26, 2010												
390 μatm	574±27	ns	106±2	ns	923±28	ns	776±12	B	234±1	C	732±18	C
750 μatm	522±24		126±33		887 ±41		698 ±20		194±4		574±19	
Aug. 28, 2010												
390 μatm	611±23	ns	130±4	ns	926±24	ns	825 ±36	A	252 ±3	C	720±35	C
750 μatm	637±17		137±35		947±45		743±23		178±6		575±15	
Mar. 14, 2011												
390 μatm	176±4	ns	119±4	ns	389±6	ns	286±6	B	272±17	A	1359 ±16	C
750 μatm	173±13		118±1		391±15		208±16		190±2		1233±22	
Mar. 16, 2011												
390 μatm	193±9	A	96±4	ns	387±11	C	305±33	ns	223±7	ns	1402±38	B
750 μatm	156±12		99±5		330±13		232±7		218±3		1158±25	

Rates of ^3^H-Leu incorporation (light and dark) incubated under both ambient (387 μatm) and elevated *p*CO_2_ (750 μatm) for both the upper (0–45 m), lower (75–125 m), and full euphotic zone (0–125 m). Significant differences between rates in controls and treatments indicated by letters (two-sample t-Test), no significant difference indicated by “ns”.

A: p<0.05.

B: p<0.01.

C: p<0.005.

## Discussion

The overarching goals of this study were to examine whether abrupt changes to the ocean carbonate system would impact organic matter productivity and bacterial growth in the NPSG. Our experiments were not designed to investigate adaptations at the gene, species, or community level. To address these objectives, two types of experiments were conducted: 1) Manipulation of the near-surface (5–25 m) seawater carbonate system by gentle bubbling with air or a mixture of air and CO_2_ and subsequent daily measurements of ^14^C-PP and ^3^H-Leu incorporation over 2 to 5 day incubation periods; and 2) Perturbation of the seawater carbonate system through the addition of acid (and bicarbonate to keep TA unchanged) at different depths throughout the euphotic zone, examining subsequent depth-dependent responses in rates of ^14^C-PP and ^3^H-Leu incorporation during *in situ* incubations lasting over the course of a photoperiod (~12 hours).

We detected no consistent changes in rates of either ^14^C-PP or ^3^H-Leu incorporation in response to elevated *p*CO_2_ over the course of our bubbling experiments. This lack of a consistent effect of enhanced *p*CO_2_ on either ^14^C-PP or ^3^H-Leu incorporation suggests that the contemporary microbial assemblages in this region of the NPSG appear relatively resilient to rapid increases in seawater *p*CO_2_. Such observations are in agreement with results from other studies conducted in oligotrophic ocean ecosystems [20,21]. However, several studies have reported small to moderate increases in rates of ^14^C-PP [7,51] and bacterial production [31] under elevated *p*CO_2_ in more eutrophic nearshore ecosystems.

Similar to the lack of response to increased *p*CO_2_ observed in the bubbling experiments, rates of production in the depth-resolved experiments also demonstrated no significant or consistent response to increases in seawater *p*CO_2_. Intriguingly, in one of our experiments (conducted in August 2010), rates of ^14^C-PP by larger phytoplankton (>2 μm) in the lower euphotic zone demonstrated greater rates of production under elevated *p*CO_2_. These dimly lit waters and larger phytoplankton size classes account for a relatively small fraction of the euphotic zone productivity in the NPSG [52], so the resulting stimulation by *p*CO_2_ resulted in no significant change in the depth-integrated (0–125 m) productivity from this experiment. Although the changes were modest (~20% and ~50%, for 2–10 μm and >10 μm, respectively), the apparent stimulation of productivity by larger phytoplankton may reflect ecological adaptations of phytoplankton in these dimly lit waters. The lower euphotic zone of the NPSG is dynamic with respect to changes in the seawater carbonate system, as a result of the large vertical gradient in DIC concentrations [5] together with the greater influence of mesoscale variability in the lower euphotic zone compared to surface waters [50]. Hence, the observed response to rapid perturbation in *p*CO_2_ could reflect an adaptive response by phytoplankton communities to abrupt changes in the seawater carbonate system in the lower euphotic zone. In addition, this observation could reflect carbon limitation of phytoplankton growing in the lower euphotic zone during summer months. Net production of oxygen (O_2_) in the sub-mixed layer waters of the NPSG results in accumulation of dissolved O_2_ throughout the spring, with supersaturating concentrations through the summer and early fall [53]. We speculate that the enhanced rates of ^14^C-PP during this single experiment may reflect alleviation of CO_2_ limitation of the larger phytoplankton growing in waters with elevated O_2_: CO_2_ ratios, where competitive binding of O_2_ by RuBisCO could decrease photosynthetic efficiency and increase photorespiration [17,54].

We also sought to examine the sensitivity of bacterial production to abrupt increases in seawater *p*CO_2_ during our depth-resolved experiments. While we observed no consistent response in rates of ^3^H-Leu_Light_ or ^3^H-Leu_Dark_ incorporation to the *p*CO_2_ treatments during the bubbling experiments, rates of ^3^H-Leu incorporation in our depth-resolved experiments were frequently sensitive to changes in *p*CO_2_. In all 5 of the depth-resolved experiments, euphotic zone (0–125 m) rates of ^3^H-Leu_Light_ incorporation were always significantly lower in the enhanced *p*CO_2_ treatments than in controls. In contrast, rates of ^3^H-Leu_Dark_ incorporation did not vary in a consistent manner, with rates of ^3^H-Leu_Dark_ incorporation greater in the controls than the *p*CO_2_ treatments in 2 of 5 experiments. We incubated samples in both the light and dark to evaluate how elevated *p*CO_2_ might alter the known photostimulation of ^3^H-Leu incorporation previously reported in the euphotic zone of the NPSG [44,55]. Based on flow cytometric sorting of picoplankton populations, Björkman et al. [56] determined that *Prochlorococcus* incorporation of ^3^H-Leu in the light was a major factor controlling this photostimulation. Given the large contribution of *Prochlorococcus* to rates of ^14^C-PP (39% ±20%) and ^3^H-Leu incorporation (20% in the dark and 60% in the light) at Station ALOHA [56,57], our results suggest *Prochlorococcus* growth may be relatively insensitive to, or perhaps negatively affected by, abrupt increases in *p*CO_2_. These results are consistent with previous findings in culture that suggest that while *Synechococcus* growth responds to elevated *p*CO_2_, *Prochlorococcus* growth appears largely insensitive to variations in *p*CO_2_ [11].

Several previous studies have reported increased rates of ^3^H-Leu_Dark_ incorporation under elevated *p*CO_2_ [10], suggesting a shift in the partitioning of primary production from the particulate to the dissolved pool [26,58,59], with subsequent increased growth by heterotrophic bacteria on this newly available DOM [31,60]. However, other studies that have specifically measured rates of dissolved organic carbon production under elevated *p*CO_2_ have reported inconsistent responses [61,62]. In our experiments, based on both bubbling and depth-resolved experiments, rates of ^3^H-Leu_Dark_ incorporation were most often unchanged under conditions of elevated *p*CO_2_. Similarly, in the near-surface waters (represented by our bubbling experiments) rates of ^3^H-Leu_Light_ incorporation were unaffected by increases in *p*CO_2_, but in the deeper regions of the euphotic zone (75–125 m), rates of ^3^H-Leu_Light_ incorporation were significantly lower under elevated *p*CO_2_ treatments relative to the controls in 4 out of 5 experiments.

In general, we found that abrupt increases in *p*CO_2_ have little or no influence on rates of ^14^C-PP and ^3^H-Leu_Light_ or ^3^H-Leu_Dark_ incorporation at Station ALOHA. On a single occasion, we did observe apparent stimulation of ^14^C-PP by larger phytoplankton dwelling in the lower euphotic zone, an observation we hypothesize could reflect seasonally-dependent carbon limitation by phytoplankton growing in these dimly lit waters. However, the majority of our experiments suggest that contemporary microbial growth in the euphotic zone at Station ALOHA is relatively resilient to abrupt increases in *p*CO_*2*_. Such results are somewhat surprising given the low temporal fluctuations in seawater *p*CO_2_ this habitat experiences; however, we suspect that in this persistently oligotrophic environment, both rates of ^14^C-PP and ^3^H-Leu incorporation are strongly controlled by the availability of growth-limiting substrates, whether in the form of inorganic nutrients or in the form of labile dissolved organic carbon. In particular, decadal-scale (2006–2016) rates of ^14^C-PP (at 25 m) at Station ALOHA varied more than threefold (from 0.28 to 0.98 μmol C L^-1^ d^-1^), approximately equivalent to the largest variation in rates of ^14^C-PP we measured between control and treatment rates during our experiments. Consequently, even large perturbations to the carbonate system appear to have only a weak influence on microbial growth in this ecosystem. Additionally, the short division times and large population sizes of open ocean phytoplankton may provide some capacity to adapt to or evolve in response to anthropogenic changes to the ocean carbonate system [63]. In contrast, it is likely that increasing ocean temperatures will exert a relatively stronger influence on microbial metabolism. Temperature effects could manifest directly, for example by changing microbial metabolic rates or growth efficiencies [64,65] or indirectly; through increased vertical stratification with concomitant reduction in nutrient supply and expansion of the oligotrophic gyres [66]. The combination of temperature-driven increases in respiration and decreased nutrient supply to the euphotic zone would likely decrease rates of net community production, with decreases in the amount of organic carbon available for upper trophic levels and export to the deep ocean. Hence, based on our observations together with those from previous reports, responses in planktonic metabolism to elevated *p*CO_2_ appear variable and likely depend on the types of organisms present and the environmental conditions under which they grow [61,67,68]. It remains an open question whether our findings reflect physiological flexibility by the resident microbial community in acclimating to changes in the carbonate system, or whether the growth of these organisms is so tightly regulated by resource availability that any influence due to variations in the carbonate system are obscured by these other controlling factors. This question could be addressed by carrying out similar perturbation experiments that examine whether microbial growth responds to elevated *p*CO_2_ coincident with alterations in the availability of growth-limiting nutrients.

## Supporting information

S1 Fig Rates of chlorophyll normalized ^14^C-PP from bubbling experiments. Chlorophyll normalized rates of ^14^C-PP from *p*CO_2_ bubbling experiments during this study where no significant difference was observed between controls (open circles) and elevated *p*CO_2_ (grey squares) treatments (panel A) and for an experiment where a significant difference was found (two-way ANOVA; p<0.05) between controls (open triangle) and elevated *p*CO_2_ (grey triangle) treatments (panel B).
(TIF)

S2 Fig Ratio of calculated *p*CO_2_ versus targeted *p*CO_2_ from *in situ* array experiments. Solid line depicts the 1:1 ratio.
(TIF)
