# Supplementary figures and images for: The influence of abrupt increases in seawater pCO2 on plankton productivity in the subtropical North Pacific Ocean
Source: PLoS One. 2018 Apr 25;13(4):e0193405. doi: 10.1371/journal.pone.0193405 (PMC5918612; doi:10.1371/journal.pone.0193405)

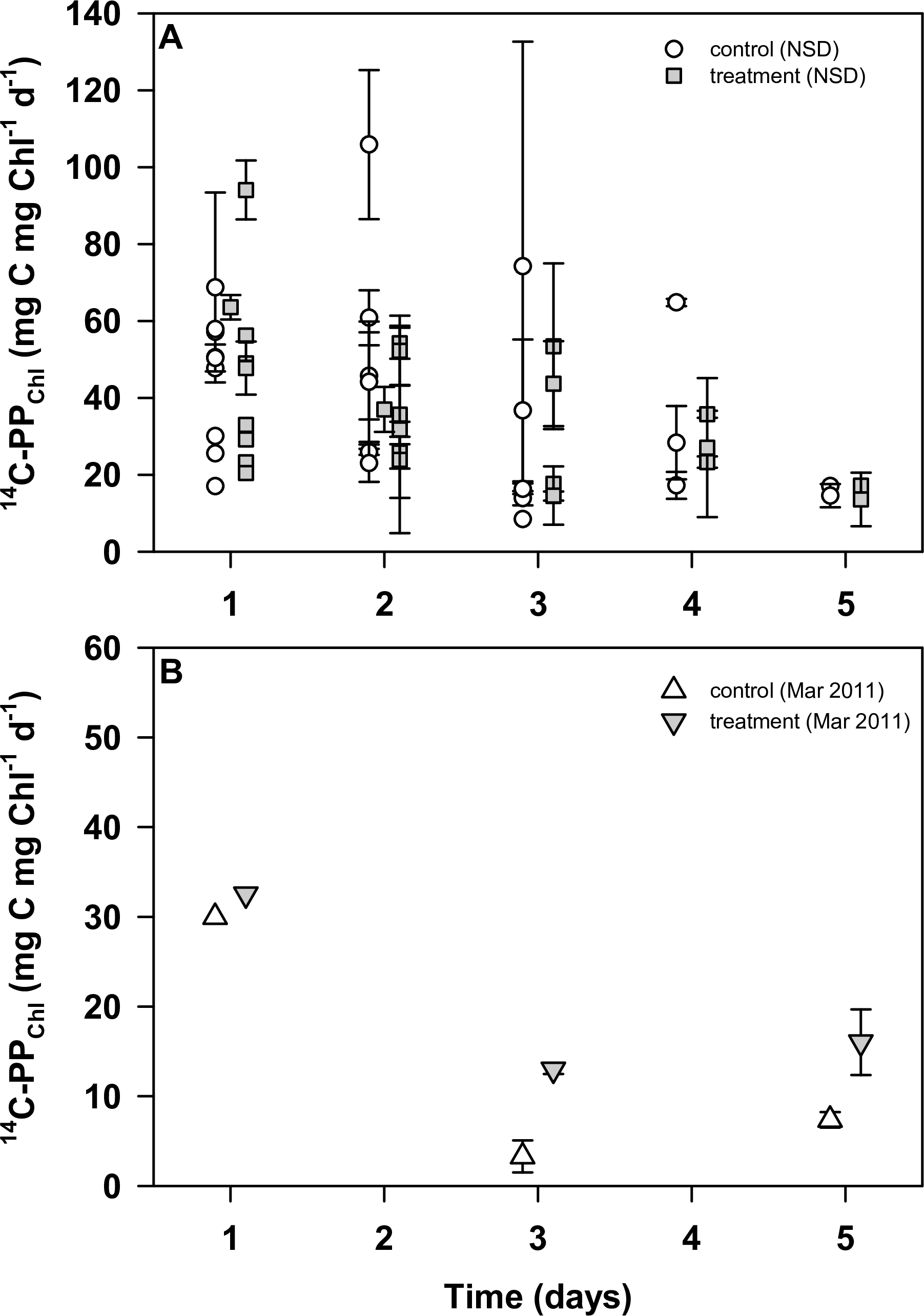

Supplement: S1 Fig — Chlorophyll normalized rates of 14C-PP from pCO2 bubbling experiments during this study where no significant difference was observed between controls (open circles) and elevated pCO2 (grey squares) treatments (panel A) and for an experiment where a significant difference was found (two-way ANOVA; p<0.05) between controls (open triangle) and elevated pCO2 (grey triangle) treatments (panel B). (TIF) [file pone.0193405.s001.tif]

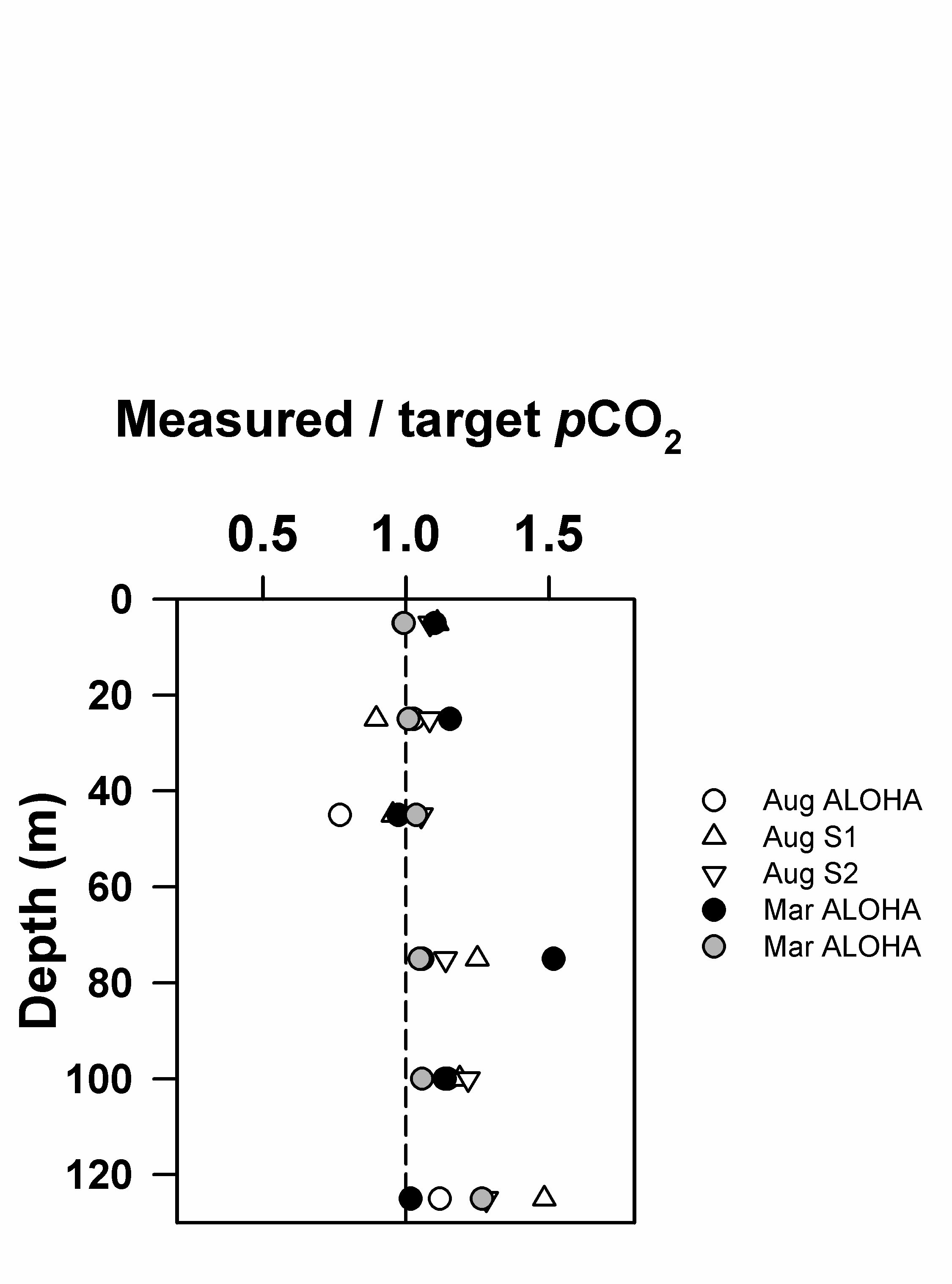

Supplement: S2 Fig — Solid line depicts the 1:1 ratio. (TIF) [file pone.0193405.s002.tif]
